# Suppression of Long-Lived Humoral Immunity Following *Borrelia burgdorferi* Infection

**DOI:** 10.1371/journal.ppat.1004976

**Published:** 2015-07-02

**Authors:** Rebecca A. Elsner, Christine J. Hastey, Kimberly J. Olsen, Nicole Baumgarth

**Affiliations:** 1 Center for Comparative Medicine, University of California, Davis, Davis, California, United States of America; 2 Microbiology Graduate Group, University of California, Davis, Davis, California, United States of America; 3 Department of Pathology, Microbiology, and Immunology, School of Veterinary Medicine, University of California, Davis, Davis, California, United States of America; Medical College of Wisconsin, UNITED STATES

## Abstract

Lyme Disease caused by infection with *Borrelia burgdorferi* is an emerging infectious disease and already by far the most common vector-borne disease in the U.S. Similar to many other infections, infection with *B*. *burgdorferi* results in strong antibody response induction, which can be used clinically as a diagnostic measure of prior exposure. However, clinical studies have shown a sometimes-precipitous decline of such antibodies shortly following antibiotic treatment, revealing a potential deficit in the host’s ability to induce and/or maintain long-term protective antibodies. This is further supported by reports of frequent repeat infections with *B*. *burgdorferi* in endemic areas. The mechanisms underlying such a lack of long-term humoral immunity, however, remain unknown. We show here that *B*. *burgdorferi* infected mice show a similar rapid disappearance of Borrelia-specific antibodies after infection and subsequent antibiotic treatment. This failure was associated with development of only short-lived germinal centers, micro-anatomical locations from which long-lived immunity originates. These showed structural abnormalities and failed to induce memory B cells and long-lived plasma cells for months after the infection, rendering the mice susceptible to reinfection with the same strain of *B*. *burgdorferi*. The inability to induce long-lived immune responses was not due to the particular nature of the immunogenic antigens of *B*. *burgdorferi*, as antibodies to both T-dependent and T-independent Borrelia antigens lacked longevity and B cell memory induction. Furthermore, influenza immunization administered at the time of *Borrelia* infection also failed to induce robust antibody responses, dramatically reducing the protective antiviral capacity of the humoral response. Collectively, these studies show that *B*. *burgdorferi*-infection results in targeted and temporary immunosuppression of the host and bring new insight into the mechanisms underlying the failure to develop long-term immunity to this emerging disease threat.

## Author Summary

Infections with the Lyme Disease agent, *Borrelia burgdorferi*, often fail to generate long-term protective immunity. We show here that this is because the immune system of the *Borrelia*-infected host generates only short-lived, structurally abnormal and non-functional germinal centers. These germinal centers fail to induce memory B cells and long-lived antibody-producing plasma cells, leaving the host susceptible to reinfection with Bb. This inability to induce long-term immunity was not due to the nature of Borrelia antigens, as even T-dependent antigens of Borrelia were unable to induce such responses. Moreover, influenza vaccine antigens, when applied during Borrelia-infection, failed to induce strong antibody responses and immune-protection from influenza challenge. This data illustrate the potent, if temporal, immune suppression induced by Borrelia-infection. Collectively, the data reveal a new mechanism by which *B*. *burgdorferi* subverts the adaptive immune response.

## Introduction

Lyme disease is the most common vector born disease in the United States and Europe [1,2]. In the U.S., the causative bacterial agent is *Borrelia burgdorferi (Bb)*. It is transmitted by *Ixodes* spp. ticks and causes a variety of clinical manifestations and sometimes debilitating disease. *Bb* requires persistence in immunocompetent vertebrate hosts, as this pathogen’s complex lifecycle requires uptake by ticks for transmission from one vertebrate host to the next [3]. *Bb* has developed multiple immune evasion mechanisms that may render antibody responses ineffective, thereby supporting ongoing infections [4]. Documented are rapid up and down-regulation of multiple highly immunogenic surface antigens during infection [5]. Antigenic variation of variable surface protein E (VlsE) seems to play a role in immune evasion [6], as does the inhibition of complement-mediated bacterial lysis [7,8]. The adaptive immune response cannot clear infection, and thus infection requires antibiotic treatment for resolution. Yet, reinfections are common in endemic areas [9–11], suggesting that *Bb* may also subvert the induction and/or maintenance of long-lived antibody responses. Although numerous studies have documented the ability of Bb to evade antibody responses, whether the antibody response is maximally induced and/or maintained has not been systematically studied.

The antibody response to *Bb* infection is complex [12]. *Bb*-tissue burden and disease symptoms are ameliorated by *Bb*-specific antibodies, which are also used for disease diagnosis [12–14]. Yet antibodies are ultimately incapable of clearing the infection [12,15]. Both sera and purified antibodies from *Bb* infected humans and mice can provide passive protection from infection in experimentally challenged mice, but protection wanes over time following antibiotic treatment [16,17], suggesting that protective adaptive immunity is not long-lived. Indeed, *Bb*-antibody titers seem to be influenced by whether the spirochetes disseminate early after infection or remain localized for a time. Slower to disseminate spirochete infections have been associated more frequently with antibody titers that suffer a precipitously decline following antibiotic treatment of patients [10,18,19].

Our previous studies demonstrated that lymph tissues are early targets of *Bb*-infection [20]. Draining lymph nodes become Bb culture positive as early as 24h after infection and Bb then spreads to the other lymph nodes, inducing strong lymph node enlargement and causing a destruction of T and B cell zones in C57BL/6 mice [20,21]. All tested lymph nodes remained Bb positive for the duration of the infection [20,22]. Germinal centers (GC) are main immune response outcomes that usually develop in secondary lymphoid tissues after infections. They are required for development of long-lived plasma cells, which provide ongoing protection by continuous antibody secretion. They also induce recirculating memory B cells, which respond quickly to reinfection with differentiation and production of high affinity antibodies [23]. Germinal centers do form in Bb-infected lymph nodes at around two week after infection, but then rapidly involute over the next two weeks [22].

Here we tested the functionality of the GC responses to *B*. *burgdorferi*. We demonstrate that following infection of mice with *Bb*, GC are structurally abnormal and long-lived plasma cells and B cell memory, normal outputs of GC responses, fail to develop for months after infection rendering mice susceptible for reinfection with the same strain of Bb. When mice were infected with *Bb*, vaccination with influenza antigens failed to induce protective anti-viral immunity, revealing that *Bb*-infection actively inhibits long-term humoral immune response development. The data provide a novel mechanism to explain the observed high rate of reinfections with *Bb* and demonstrate that acute *Bb*-infection induces a transient state of immune suppression.

## Results

### Failure to produce long-lived antibodies to a T-dependent Borrelia antigen

Previous clinical studies have suggested that the antibody responses to Bb might fail to be induced long-term, as patients treated with antibiotics lose measurable Borrelia-specific serum IgG [18]. We had shown previously that Bb rapidly targets lymph nodes [20–22], the induction sites of immune responses, suggesting a possible connection between the observed lack of strong immune response induction and the rapid infections of the lymph nodes.

**Fig 1:**
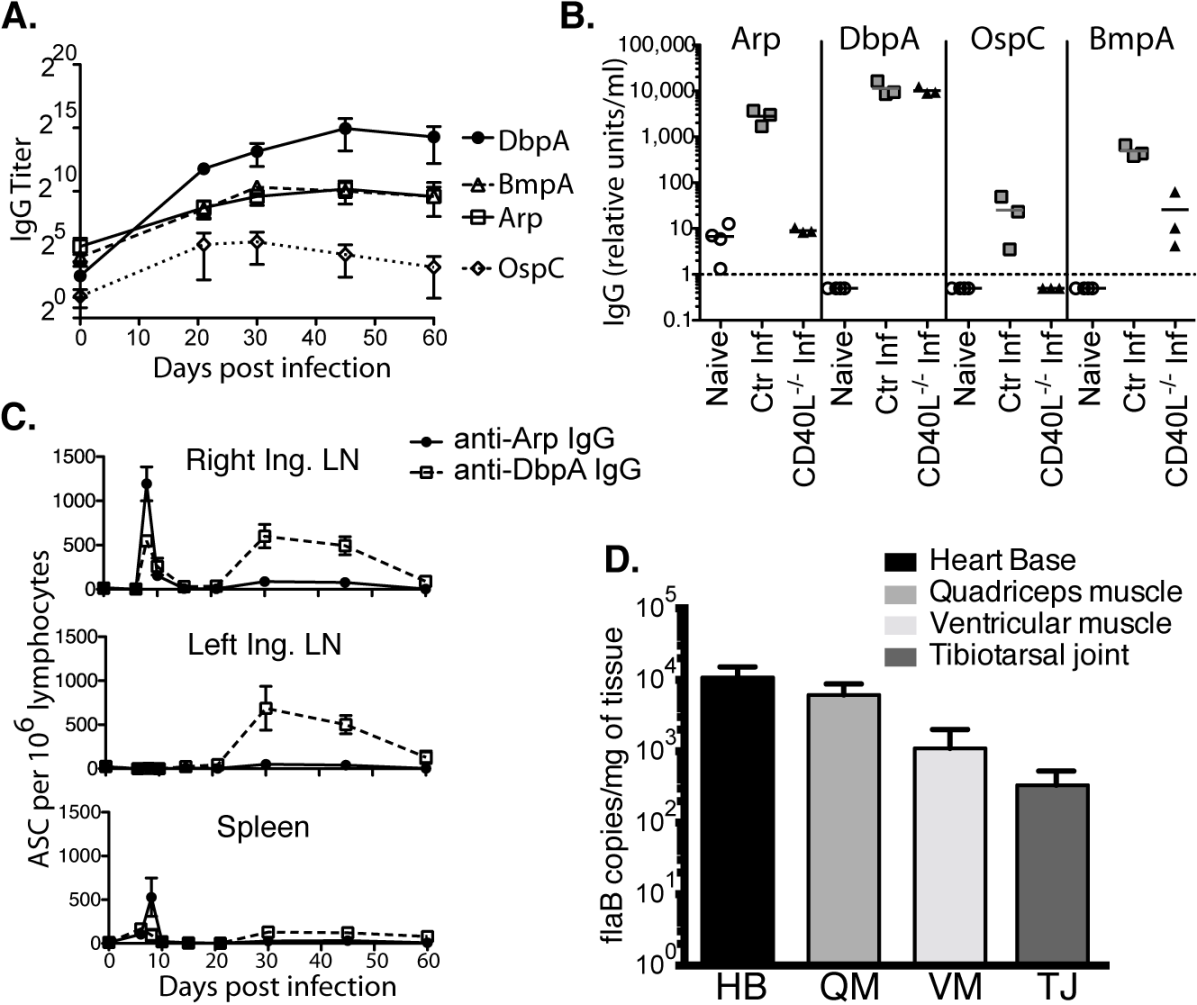
Induction of T-dependent and T-independent antibody responses to long-term Bb infection. **(A)** Reciprocal endpoint titer of serum antigen-specific IgG was determined at the indicated times post infection (mean ± SD, n = 4 per time point). One representative time course of two is shown. **(B)** Serum antigen-specific IgG in CD40L -/- and B6 controls at day 120 of infection (n = 4 per group). Naïve (but not age matched) controls were included for comparison. Symbols represent individual mice, bars indicate mean for the group, and the dashed bar represents the limit of detection. Samples below the limit of detection were arbitrarily assigned a value of ½ the limit of detection. Results from one representative experiment of two are shown. **(C)** Mean frequencies (n = 4 per time point) ± SD of Arp- and DbpA-specific antibody secreting cells (ASC) as quantified by ELISPOT. Results were from two experiments, one for days 0, 6, 8, 10, and 15, and another for days 21, 30, 45, and 60. **(D)** Shown are mean ± SD Bb copy numbers as measured by qPCR in indicated tissues of C57BL/6 mice (n = 6) 14 months after infection with Bb N40.

T-independent antigens, such as lipids and carbohydrates induce only short-lived antibodies, which are expected to decline following removal of the pathogen, while T-dependent antibodies would be expected to induce a long-term response. In order to determine the nature of the antibody responses to Bb, we measured the induction and kinetics of *Bb*-specific antibody responses to a set of previously identified strongly immunogenic antigens [20]. Antibodies against arthritis-related protein (Arp), decorin-binding protein A (DbpA), outer-surface protein C (OspC), and the outer surface protein BmpA of cN40 *Bb* are not only highly immunogenic, they also passively protect from infection or are involved with resolution of arthritis and carditis, or both [24–27]. We found induction of IgG against each in C57BL/6 mice (Fig 1A). Only OspC- and Arp- but not DbpA-specific IgG responses required the presence of CD40L, thus identifying DbpA as a T-independent and OspC and Arp as T-dependent antigens (Fig 1B), consistent with previous studies [13,28]. Given the high antibody titers against Arp, and the complete dependence on CD40/CD40L interaction, this response served as a measure of T-dependent extrafollicular and/or GC responses, while the strong anti-DbpA IgG response was used as a measure of prototypic T-independent extrafollicular responses. DbpA-specific, but not Arp-specific antibodies, were also unaffected by removal of CD4 T cells for 60-days [28].

Kinetic analysis of IgG antibody-secreting cells (ASC) to Arp and DbpA demonstrated their peak induction in the draining lymph nodes and spleens at day 8 of *Bb*-infection (Fig 1C). A second peak composed mostly of T-independent DbpA-specific IgG in both lymph nodes followed about 3 weeks later, but by day 60 ASC frequencies had dropped to pre-infection levels in all tissues (Fig 1C). Given that GC responses did not develop before day 15 ([29] and see below), the early peak response to both T-dependent and T-independent *Bb* antigens is likely the output of the earlier-induced extrafollicular B cell responses [30].

**Fig 2:**
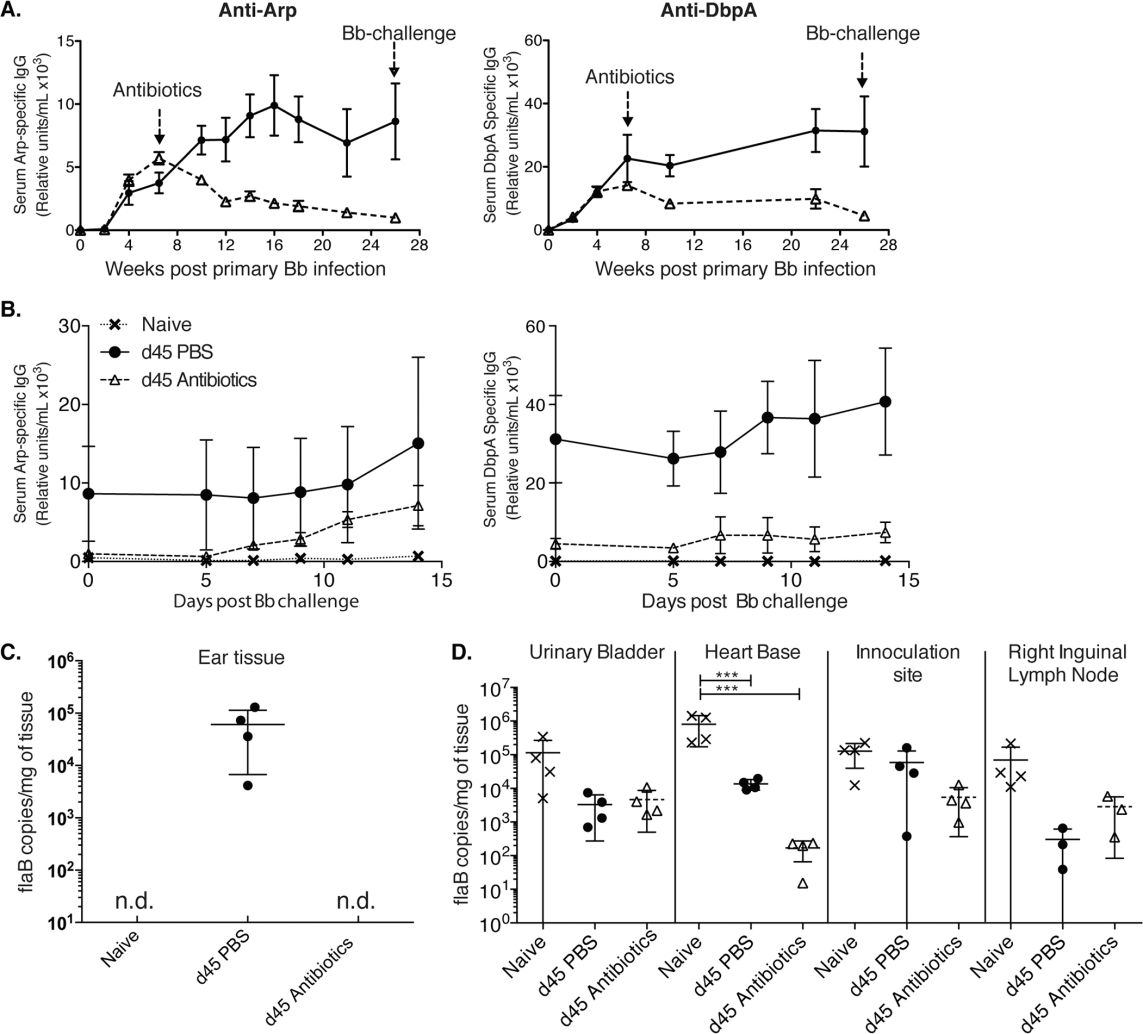
Lack of long-lived antibody responses after Bb infection (A-B) Shown are relative units Arp- (left) and DbpA- (right) specific serum IgG ± SD (n = 4 mice per group) as assessed by ELISA before and after daily treatments with ceftriaxone for 10 days (grey dashed line) starting at day 45 of infection with *Bb*. Controls received PBS treatment only (black solid line). (**B**) Re-challenge using the same Bb strain (N40) of mice at twenty-six weeks after initial Bb infection mice induced rises in anti-Arp-specific antibody titers. (**C**) Shown are mean +/- SD of Bb FlaB DNA copy numbers in ear tissues of indicated groups of mice at 26 weeks after primary infection, just prior to Bb-challenge. (**D**) Bb-tissue loads of mice at day 14 post challenge as assessed by qPCR. Significance was determined by two-way ANOVA Tukey’s multiple comparisons test. *** p < 0.0001. N.d. not detected.

C57BL/6 mice are persistently infected with Bb N40, as shown by the strong presence of Bb DNA in all tested tissues of mice 14 months after initial infection (Fig 1D). Thus, the continued production of antibodies could be due either to an ongoing induction of short-lived plasma cell responses, or due to the development of long-lived plasma cells. The latter is the typical outcome of an infection and known to be induced in GC responses [31]. To measure the functional capacity of *Bb*-infection to induce long-lived antibody-secreting plasma cells, we studied maintenance of serum antibody responses over the course of long-term infection. As expected serum antibody responses to both Arp and DbpA were strongly induced after infection. However, following resolution of infection via antibiotic treatment, which was initiated 6 weeks after infection, antibody responses to both Arp and DbpA declined significantly beginning almost immediately after treatment (Fig 2A), demonstrating a failure of the immune system to generate long-lived plasma cells, including to a T-dependent antigen of *Bb*.

### Susceptibility of Bb-infected mice to reinfection with the same strain of Bb following antibiotic treatment

Indeed, a previous report showed a lack of plasma cell accumulation in the bone marrow for months after *Bb* infection [22] and humans treated with antibiotics often show a rapid decline of *Bb*-specific serum antibodies, which can provide challenges regarding serological diagnosis of *Bb*-infection [10,18,19]. Thus, the mouse faithfully recapitulates the clinically observed drop in antibody-titers after antibiotic treatment, even when this treatment is initiated six weeks after infection. The here observed lack of long-term antibody production after antibiotic treatment suggested that mice might become susceptible to challenge with the same strain of Bb after Bb-specific antibodies, generated only by short-lived plasma cells during active infection, have decayed. To test this, we followed the very strong Arp and DbpA-specific antibody titers in mice infected with Bb and treated or not with antibiotics on day 45 after primary infection until their antibody levels reached near pre-infection levels. This occurred at about 26 weeks after initial infection (Fig 2A). At that time point both groups of mice and a third group of non-infected mice were challenged with the same strain of Bb used for the primary infection (N40). In antibiotic-treated mice serum-antibody levels to Arp showed strong induction within 7 days of challenge (Fig 2B). Antibody levels to DbpA also increased, but only slightly. The responses were stronger than those measured in naïve mice, suggesting the presence of residual immunity that can be boosted at this 6 months time point.

To determine whether mice were not just immunologically responsive to a secondary infection but were susceptible to Bb reinfection, we determined Bb tissue loads by culture and qPCR. Urinary bladder tissues of four control mice infected with Bb and treated for 10 days with ceftriaxone were all Bb negative on day 62 after infection, i.e. one week after end of antibiotic treatment, while the non-antibiotic treated mice were all culture positive. In addition, qPCR analysis of ear tissue at week 26 after infection just prior to Bb-challenge, demonstrated that only Bb-infected and non-treated mice were Bb-positive by qPCR, whereas the antibiotic-treated group and as expected, the naïve mice, were Bb DNA negative (Fig 2C). In contrast when multiple tissues were harvested and tested for Bb DNA all tissues from all groups were DNA positive 14 days after Bb challenge (Fig 2D). There was no statistical significant difference in Bb-load in the three tested tissues from challenged mice either treated or not with antibiotics at that time point. Naïve mice had a significant higher Bb-load in the heart-base compared to the two previously infected groups (p < 0.0001). Thus, the data indicate that the failure to induce long-lived plasma cells renders mice previously infected and then treated with antibiotics susceptible to reinfection with the same strain of Bb when sufficient time has elapsed so that antibody-levels have dropped close to pre-infection levels.

### Bb infection induces short-lived germinal centers with little complement deposition

**Fig 3:**
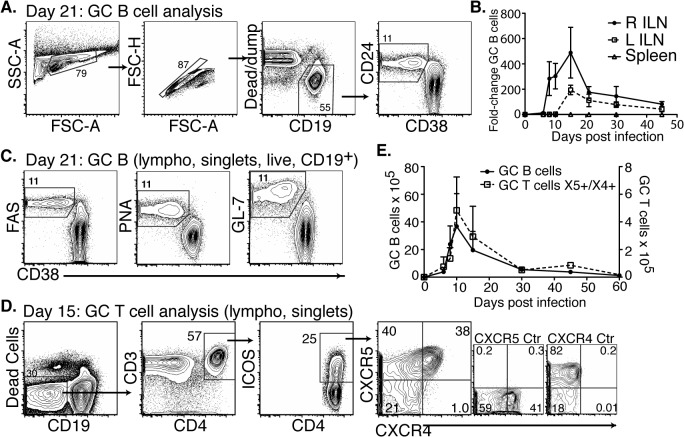
CD4 T_FH_ and GC B cells develop after *Bb* infection but responses are short-lived. **(A-C)** Right and left inguinal lymph nodes (IngLN) and spleens were analyzed by flow cytometry at the indicated times after *Bb* infection of B6 mice (n = 4 per time-point). Data were compiled from two time-course studies (days 0, 6, 8, 10, 15, and 30 and days 21, 30, and 45, respectively). **(A)** 5% contour plots with outliers showing gating for germinal center (GC) B cells on a representative sample at day 21 post-infection. **(B)** Mean total numbers ± SD GC B cells. **(C)** Analysis of additional GC B cell markers, representative samples are shown from day 21 post-infection. **(D-E)** Mice (n = 4 per time-point) were infected and right IngLN were collected for FACS analysis (D and E). **(D)** 5% contour plots with outliers of gating for GC T cells. **(E)** Mean total numbers ± SD of GC B (left y-axis) and T cells (right y-axis).

T-dependent long-term antibody responses are the outputs of germinal centers (GC), which are generated in secondary lymphoid tissues draining sites of infection. Therefore, we focused next on the development of GC during Bb infection. As shown previously, infection of mice with host-adapted spirochetes into the right hind-leg resulted in strong responses in all lymph nodes, with the right and left inguinal lymph nodes responding first and last, respectively, in the order in which they become Bb-culture positive [20]. Bacteria are also eventually found in the spleen, but at much lower levels [20]. Consistent with previously observed kinetics, GC B cells (CD19^+^CD24^hi^CD38^low^, PNA^+^, FAS^+^, and GL7^+^ Fig 3A and 3C) were induced following *Bb* infection, peaking around day 15 and quickly declining to near pre-infection levels by day 45 in all studied lymphoid tissues (Fig 3B). Few GC B cells were found in the spleens at any time after infection (Fig 3B). The rapid decline of GC B cells in all tissues, despite the ongoing infection was consistent with the lack of long-term antibody production observed above (Fig 2) and suggested that GC responses to Bb might be defective.

**Fig 4:**
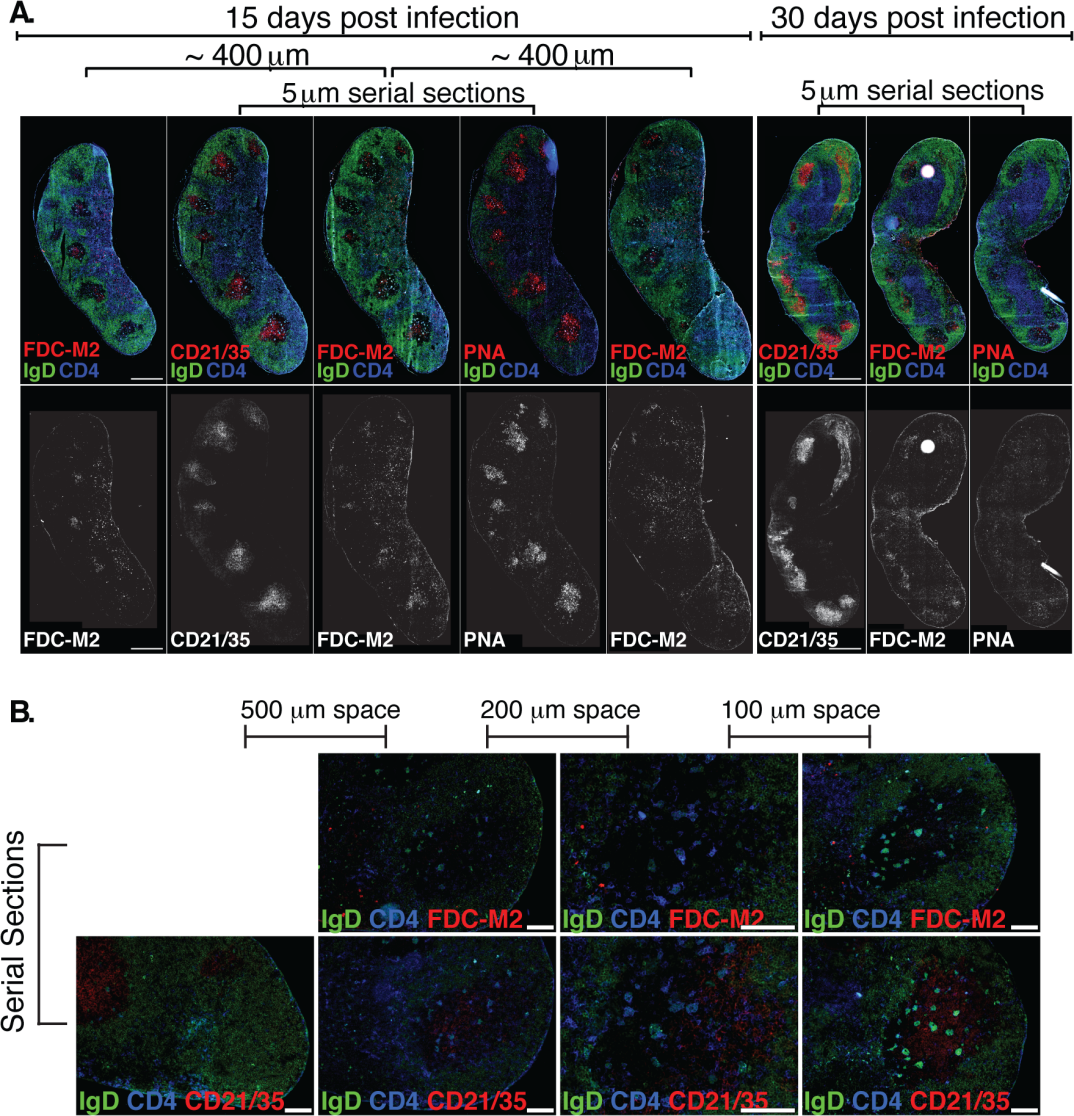
(A) Left Inguinal (Ing) LN from Bb-infected mice were analyzed by immunofluorescence for follicular dendritic cell markers FDC-M2, CD21/35, and for germinal centers (PNA; all red). Shown is staining on serial sections at days 15 and 30 post-infection (n = 2 each time point). Sets of 3 serial sections were prepared every ~400 μm to view multiple planes of each GC. Shown is one representative set of serial sections from days 15 and 30, and additional FDC-M2 stained sections flanking the center set for day 15. IgD staining indicates B cell follicles (IgD+, green) and GC (IgD- within follicles). CD4 T cells (blue) can be seen within GC. **(B)** At day 15 of Bb infection, serial sections were prepared to analyze FDC-M2 (top panel, red) and CD21/35 (bottom panel, red) expression on germinal center follicular dendritic cells (n = 2). IgD staining indicates locations of both B cell follicles (IgD+, green) and germinal centers (IgD- within IgD+ follicles). CD4 T cells (blue) can be seen within germinal centers. Sets of serial sections were prepared at approximately 100–500 μm intervals as indicated. Images third from the left were captured at 20x, all others at 10x, and all scale bars represent 100 μm.

Two additional cell populations are critical for the functionality of GC: T follicular helper cells (T_FH_), and follicular dendritic cells (FDC) [32]. T_FH_ cells provide crucial signals to GC B cells, ensuring ongoing proliferation and affinity-maturation. Their responses have not previously been studied in Bb infection. We found that T_FH_ and specifically CD4^+^ICOS^+^CXCR5^+^CXCR4^+^ GC positioned T cells [33] did develop after *Bb* infection (Fig 3D). They showed a similar rise and rapid decline to nearly pre-infection numbers by day 45 of infection than GC B cells (Fig 3E). Immunofluorescence examination of lymph nodes confirmed that GC contained CD4 T cells (Fig 4), suggesting that T_FH_ development and positioning occurred after Bb infection.

The FDC network is identified by their morphology, position within the GC, high expression of complement receptors CD21/35 and staining with the classical “FDC marker” FDC-M2, which is now understood to identify the complement component C4, deposited on the cell surface of FDC [34]. It functions to membrane-anchor antigens for effective presentation to GC B cells. Immunofluorescence staining of lymph nodes of mice immunized with a model antigen (influenza virus) in adjuvant reveals the robust induction of germinal centers, whose FDC network is strongly stained with FDC-M2 (S1 Fig). In contrast, similar analysis of lymph nodes from *Bb*-infected mice remarkably showed only weak FDC-M2 staining (Fig 4A–4B and S1 Fig). In addition, some GC showed unusual positioning of the FDC network, failing to position opposite the T cell zone (Fig 4B and S1 Fig), as seen after immunization ([35], S1 Fig). Furthermore, staining with PNA, a marker for germinal center cells was nearly absent from GC by day 30 of infection, underscoring the strong and complete involution of GC within weeks after first infection. Together the data indicate that while T and B cell responses are induced, structural deficits in FDC stromal cells are apparent and provide further evidence that Bb infection causes the structural disintegration of lymph nodes in infected C57BL/6 mice [20]. These deficits in the FDC network might inhibit the maintenance of GC responses during *Bb*-infection. Together this data demonstrate that germinal center responses are short-lived and structurally abnormal, suggesting that the failure to induce long-term antibody responses after Bb-infection is the outcome of a lack of functional GC response induction.

### Early germinal centers systemically fail to produce antigen-specific memory B cells

**Fig 5:**
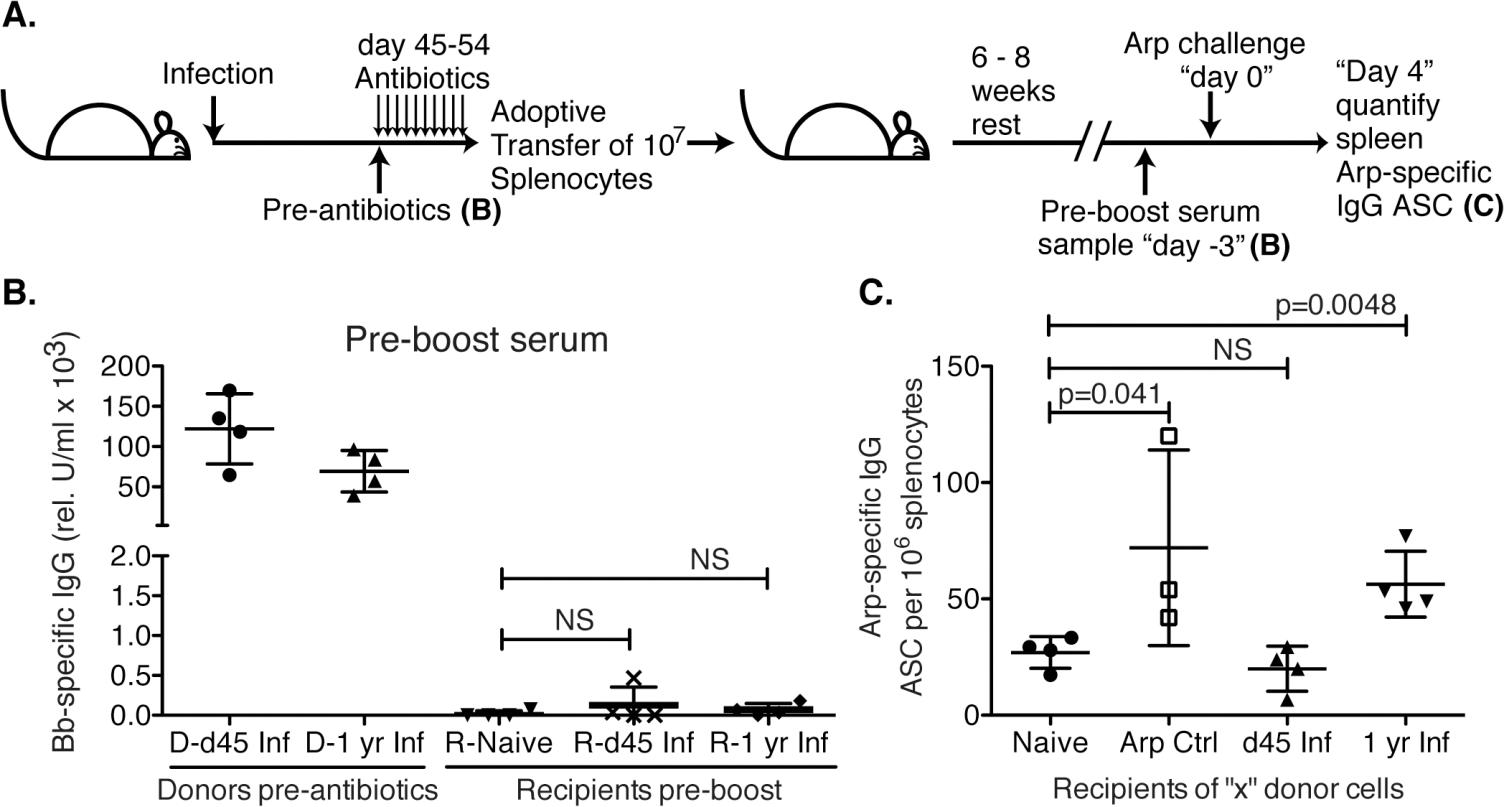
Lack of B cell memory induction after *Bb* infection. **(A)** B6 mice (n = 4 per group) were infected with *B*. *burgdorferi* and treated once daily with ceftriaxone for 10 days beginning on day 45 or 1 year post infection. One day after completion of treatments, splenocytes from *Bb-*infected mice were adoptively transferred into naïve recipient mice. Transfer of splenocytes from naïve and recombinant Arp immunized donor mice served as negative and positive controls, respectively. **(B)**. Shown are relative units serum IgG to *Bb*-antigens for donors at the time of transfer, and recipients 6–8 weeks after adoptive transfer, prior to antigen-boost. All recipients remained seronegative for *B*. *burgdorferi*-specific serum antibodies to Arp, OspC, DbpA and BmpA. Significance was determined by 1-way ANOVA. **(C)** Shown are mean frequencies ± SD *Bb*-specific IgG antibody secreting cells (ASC) in the spleens 4 days following boost as enumerated by ELISPOT. Points represent individual recipients (n = 4), and P values were calculated by Student’s t-Test in comparison to the naïve control. Results from one representative of two experiments.

GC responses lead to the development of memory B cells, which respond rapidly to reinfection by seeding new germinal centers and/or differentiating to ASC. To further assess the functionality of the GC response we studied memory B cell induction following *Bb*-infection. For that we adoptively transferred spleen cells from mice infected for 45 days or 1 year into naïve recipients, as a potential source of recirculating memory B cells (Fig 5A). To avoid transfer of spirochetes from infected donor mice, donor mice were treated with antibiotics for 10 days prior to cell transfer. Serum analysis of recipient mice confirmed the absence of significant levels of *Bb*-specific IgG and thus infection-induced primary immune responses (Fig 5B). Controls received splenocytes from either naïve (negative) or recombinant (r) Arp-immunized (positive control) donors. Six–eight weeks after transfer mice were immunized i.v. with rArp to induce differentiation of memory B cells to ASC. Arp-specific ASC were quantified in spleens 4 days later. The results demonstrated a complete failure of *Bb*-infected mice to generate memory to the T-dependent antigen Arp within the first 6 weeks of infection, as recipients of splenocytes from day 45 infected mice showed no significant induction of Arp-specific AFC from any transferred memory B cells, compared to recipients of splenocytes from naïve mice, while those of rArp-immunized mice showed a significant increase (Fig 5C). Memory B cell development was not completely prevented, but rather delayed, as Arp-specific memory B cell responses were observed in mice infected with *Bb* for one year (Fig 5C). Repeated attempts to generate memory B cell responses to DbpA by immunizing mice with recombinant protein or by infecting mice with Bb and then performing adoptive transfer failed to provide any evidence of DbpA-specific memory infection, further supporting the T-independent nature of the antibody response to DbpA (Fig 1). We conclude that following *Bb*-infection functional GC responses are not induced for many months after infection, resulting in a lack of long-lived antibody responses and the development of B cell memory.

### Bb infection actively suppresses induction of humoral immunity to an unrelated antigen

**Fig 6:**
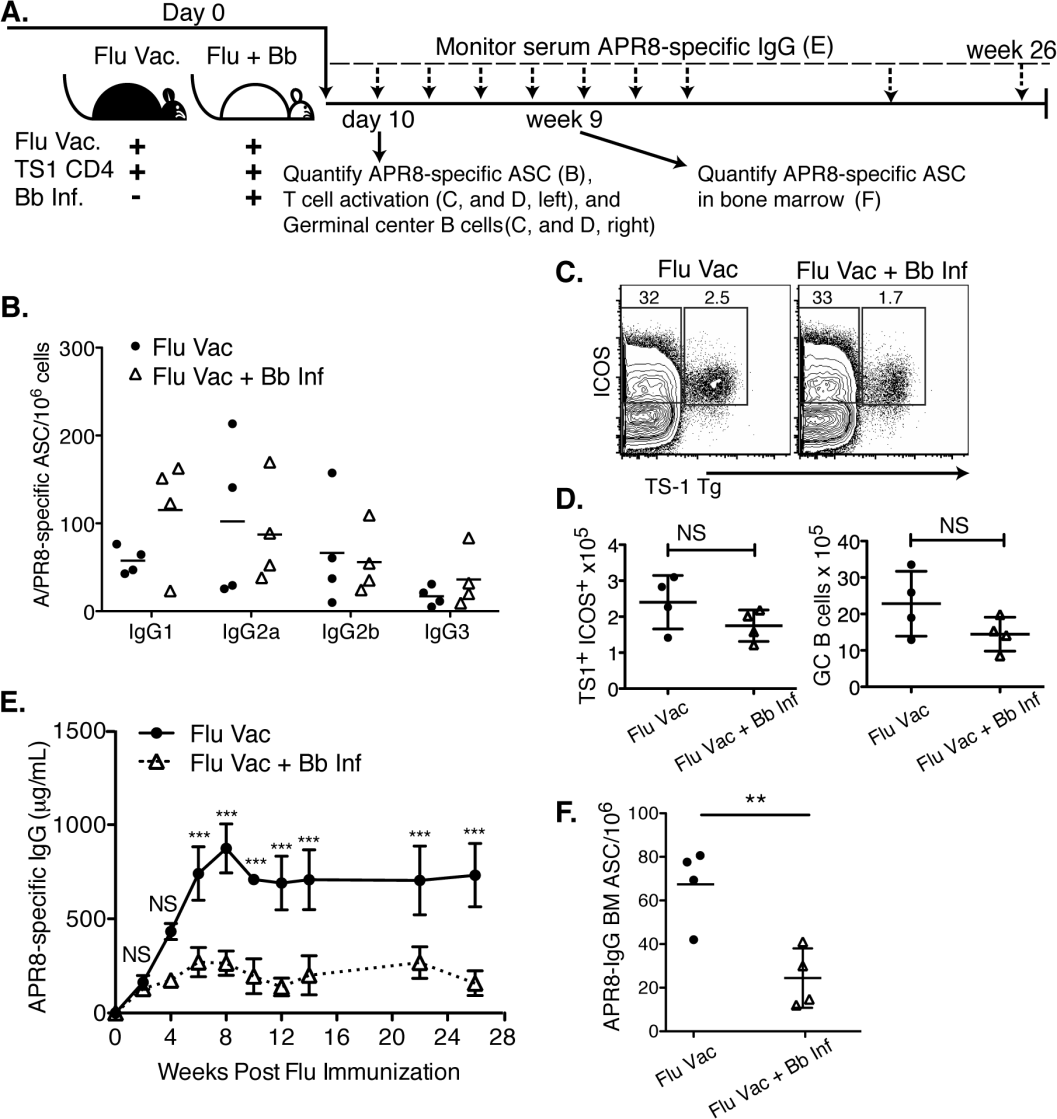
Infection with *B*. *burgdorferi* suppresses the generation of protective IgG responses to influenza vaccination. **(A)** Experimental outline. Two groups of BALB/cByJ mice (n = 4 per group) were vaccinated s.c. with influenza A/PR8 and received 1x10^6^ transgenic CD4^+^ cells specific for influenza A/PR8 HA. One group was infected with *Bb*. **(B-D)** At day 10, right inguinal lymph nodes were analyzed by **(B)** ELISPOT to quantify APR8-specific ASC, **(C, D)** and flow cytometry for HA-specific CD4 T cell activation and GC B cells. **(B)** No significant differences in A/PR8-specific antibody secreting cells were observed for individual IgG-isotypes as calculated by Student’s t-Test. **(C)** Representative FACS plots for transgenic T cell analysis, and **(D)** mean total numbers of ICOS+ HA-specific T cells (left), as well as GC B cells (right). Significance was calculated by Student’s t-Test. Results are from one experiment of three. **(E)** Mean concentration ± SD (n = 4) of A/PR8 specific serum IgG. Statistical significance was calculated using 2-way ANOVA and samples were matched over time. Results are from one experiment of two. **(F)** Mean numbers ± SD bone marrow A/PR8-specific IgG ASC quantified by ELISPOT 9 weeks post infection. Significance was calculated by Student’s *t*-Test and data are from one experiment.

These findings suggested that the host is capable of producing memory to *Bb* antigens, but that memory formation is suppressed for months after initial infection. To determine whether this lack of long-term immune induction was due to the nature of the Bb-antigens, their expression or ability to induce T cell help, or the outcome of infection-induced active immunosuppression of effective GC responses, we investigated the effects of *Bb*-infection on the B cell response to a co-administered vaccine antigen. For that, we infected mice or not with *Bb* into the right hind leg, and then vaccinated the mice simultaneously with influenza A/Puerto Rico/8/34 (A/PR8) in alum at the base of the tail. To follow antigen-specific T cell responses, mice received in addition 1x10^6^ transgenic CD4^+^ T cells (TS-1) specific for A/PR8 hemagglutinin peptide 110–119 (Fig 6A).

Consistent with the strong induction of early extrafollicular B cell responses to Bb-infection, no significant differences in the magnitude or isotype-profile were observed for influenza-specific ASC IgG antibody production in the lymph nodes on day 10 after infection between vaccinated and vaccinated and *Bb*-infected mice, as determined by ELISPOT analysis (Fig 6B). The frequencies and absolute numbers of activated HA-specific (TS1^+^) CD4^+^ T cells, as measured by induction of ICOS, a co-stimulatory molecule critical for GC B cell responses, was also similar in both groups of mice (Fig 6C and 6D). And finally, serum IgG responses to A/PR8 over the first 3 weeks of infection showed no difference compared to the vaccine-treated only mouse group. Thus the early antibody response to both Bb and to the co-applied vaccine appeared strong.

However, dramatic differences in the antibody responses to the influenza vaccination became apparent by about four weeks of infection. A/PR8-specific IgG responses plateaued around this time in the *Bb*-infected group, while they continued to rise in the vaccinated-only group, resulting in serum IgG levels at that were greatly higher compared to the titers seen in the Bb-infected and vaccinated group after 4 weeks until study end (26 weeks; Fig 6E). The lack of serum IgG responses in the Bb-infected mice might have been due to the reduction in bone marrow ASC by 9 weeks of vaccination/infection compared to the vaccine-only group (Fig 6F).

**Fig 7:**
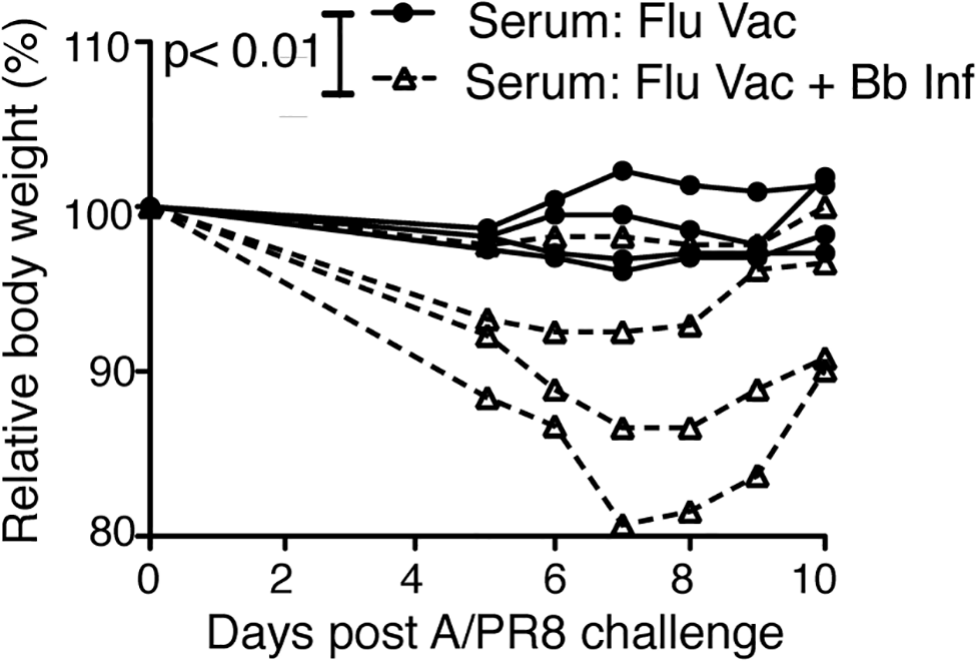
Vaccination of Bb-infected mice fails to induce protective anti-viral antibody responses Serum from BALB/cByJ mice vaccinated s.c. with influenza A/PR8 and either infected or not with Bb was harvested at 9 weeks after vaccination/infection and transferred i.v into individual recipient mice. Shown is % weight loss of individual recipients as a measure of protective capacity of the serum after i.v. serum adoptive transfer and i.n. infection with A/PR8. Each line represents one mouse, which was the recipient of serum from one donor mouse. P<0.01 was determined using paired one-tailed Student’s *t* test.

To determine whether the Bb-infection-induced changes to the B cell response induced to the influenza vaccination had a functional effect on the protective capacity of the vaccine, we conducted passive protection experiments. Remarkably, when naïve mice received immune serum from mice either Bb-infected or not and then vaccinated with influenza antigens, only mice receiving serum from flu-vaccine-only mice were protective from clinical signs of disease after influenza virus challenge (Fig 7). We conclude that *Bb*-infection suppresses the induction of long-term protective immunity to a co-administered vaccine antigen.

## Discussion

Our study demonstrates that Bb infection of mice causes a state of temporary immunosuppression that targets the hallmarks of adaptive immunity: its memory response. The lack of long-term antibody induction and memory formation was not due to previously identified immune evasion strategies of Bb, such as the down-regulation of immunogenic surface antigens, or the nature of the Borrelia antigens, such as high levels of lipidation or carbohydrate modifications, but affected responses even to co-administered highly immunogenic T-dependent influenza protein.

The mouse model faithfully recapitulated serological observations made in humans with confirmed cases of Bb infections, which noted a loss of serological signs of prior exposure after antibiotic treatment [18]. Similarly, a study in dogs showed that antibody levels rise during the summer months and then fall off considerably during the winter months when tick-activity is low, although they remained above the threshold of detection set with sera from dogs that lacked prior exposure. In each subsequent year of life antibody-titers to Bb increased, but only in animals living in endemic areas, suggesting that reinfections were common, although the infecting Bb strains responsible were not isolated [36]. Given that the antibody profile in dogs appears to be quite similar to that of humans and mice [37], studies on naturally infected dogs might provide a good model system of natural infection for future investigation on the potential epidemiological impacts of falling antibody levels.

The here shown data may also explain the previously described loss of protective serum antibodies after early antibiotic treatment, in humans and mice [16,17]. It is likely, however, that it is not just the quantity but also the quality of the antibody response that changes over the course of infection. The eventual appearance of long-lived responses, demonstrated by the accumulation of bone marrow plasma cells after day 100 of infection [22] and the presence of Arp-specific memory B cells after one year of infection (Fig 5C), suggests that the ongoing Bb infection continuously shapes and reshapes the host immune response, and that later-exerted immune-mediated control of the bacteria overcomes some of the early-induced immunosuppressive effects of the infection. It is tempting to speculate that *Bb* requires suppression of early-induced GC B cell responses, but does not require its continued suppression for fulfillment of its lifecycle. Our studies do not speak to the nature of such differences between *Bb* in early versus later stages of infection. However, GC B cell responses not only result in longer-lasting responses, they usually also result in increased antibody-affinity for their pathogen targets. As we recently demonstrated, anti-Arp antibody affinity maturation was measurable during the first 4–6 weeks of infection, but was not sustained, such that by 12 weeks of infection affinity of Arp-specific serum antibodies were similar to those 2 weeks after infection [28]. It will be important to determine whether restoring GC responses during Bb-infection would result in anti-*Bb* antibodies of higher affinity and whether these would have a higher potential to clear the infection.

The studies here focused on B cell responses to Bb induced in the lymph nodes, as we found Bb spirochetes by culture and by histology as early as 24h after infection [20]. While the lymph nodes then remained culture positive throughout the infection, the spleen was largely culture negative [20]. The reasons for this lack of splenic involvement during disseminated systemic Bb infection that would be expected to result in antigen presentation and immune response induction also in the spleen are unclear. Interestingly, a study on *Ehrlichia muris* infection, another tick-transmitted disease, similarly noted the absence of splenic involvement in the immune response to both *E*. *muris* and a co-administered model antigen, but found lymph node responses to be unaffected [38].

From these results we propose a model of Bb immune evasion in which the accumulation of live Bb in lymph nodes causes changes in tissue structure and a rapid expansion of their cellularity [20,21]. The presence of Bb, which is known to express numerous complement-inhibitory proteins [7,8], including a C4b-binding protein [39], or the ensuing inflammation of these tissues, may result in a reduction in local complement C4 deposition on the FDC, which is indicated by the lack of staining for C4 deposition on FDC (Fig 4 and S1 Fig). While speculative at this time, it is possible that a lack of antigen-C4 deposition diminishes antigen-presentation by the FDC to GC B cells. Reduced antigen-presentation could result in the premature collapse of the GC responses, which we have demonstrated here and previously [20,22,28]. We hope to test this model in the future.

The results of this study may impact the interpretation of current efforts to enhance serological testing for Lyme disease. Should humans fail to generate long-lived plasma cells and memory B cells, similar to what we demonstrate here for mice, then serological testing for antibodies to Bb as a means to assess prior exposure rates is a strategy of limited scope. This would be true even if such assays were improved, such as the use of recombinant proteins generated from highly immunogenic Bb proteins, because the rate of positivity for a given test would not be affected by exposure rates but rather by the time between the last Bb exposure and testing. Previous findings in patients, showing that disseminated disease or long-term infection prior to diagnosis and antibiotic treatment are correlated with higher serological responses, are consistent with the here reported findings in mice [18,19]. However, we also note that robust IgG responses to the four here used recombinant Bb antigens were measurable in the serum of mice starting around day 15 of infection [22] and continue to be present for as long as the infection remained (Fig 2). Antibody responses were detectable significantly earlier when lymph nodes were harvested for ELISPOT analysis, around days 7/8 of infection [20,22]. Biopsy of a lymph node for early serological detection of infection is obviously not a feasible clinical approach, but the generation of early plasma blast responses after Bb infection might be measurable in the blood and has been used in other infections to assess effective immune induction [40]. The main limitation of this approach is the usually tight window, spanning only 1–2 days, in which such cells are present at measurable numbers in the blood.

Our studies in mice may provide impetus for systematic clinical studies of long-lived immunity to *Bb* in humans. The herein described immune-suppressive effect of *Bb*-infection might be considered as a rationale for testing vaccine-efficacy to unrelated antigens in Bb-infected humans or animals. It may also affect the pathogenesis of co-infections transmitted via the same tick-bite, an area of emerging interest [41,42]. A recent study reported that 1.5% and 7.8% of nymph and adult Ixodes spp. ticks, respectively, sampled in upstate New York were co-infected with *Anaplasma phagocytophilum* or *Babesia microti* [43]. Consistent with that report, 4% and 22% of patients, respectively, suspected of a tick-borne infection were co-infected with either *Bb* and Anaplasma or *Bb* and Babesia [41]. Increased susceptibility of *Bb*-infected individuals to co-infections is indicated by data provided in this study and should encourage vigorous clinical testing.

Considering the epidemiology of Bb, the suppression of long-term immunity could serve to maintain a pool of susceptible individuals, even in highly endemic areas. Based on the current literature an our data, it appears that when reinfection with Bb occur shortly after primary infection, while antibody titers and passive protective capacity are still high, such infections might preferentially be due to different strains of *Bb*, or *Bb* expressing different variable surface antigens and thus are able to evade existing immunity. However, as antibody protective capacity wanes due to a lack of long-lived immunity and declining antibody titers, individuals would be vulnerable to reinfection independently of the degree of similarity of the newly encountered *Bb* strain with the original Bb strain. A recent study by Nadelman et al [9] followed human patients that showed recurrent skin lesions, erythema migrants, which is indicative of infection with Bb. In 16 of 17 patients Bb cultured from these patients showed that the infecting Bb strain was distinct from that of the initial infection as measured by sequencing the polymorphic OspC gene. One patient showed signs of repeat infection with the same strain of Bb. The data thus support our findings that at least some human patients remain vulnerable to reinfection with the same Bb strain. Whether the others were not exposed to the same strain of Bb, or where infected with a distinct strain because of protective pre-existing antibody levels to the original strain are important questions that remain to be addressed in the future.

By identifying the GC response as a target of Bb infection-induced immunosuppression, we have opened the field to a new set of questions, including what are the molecular targets of this suppression and what are the Bb-antigens that are driving these effects? Answering these important questions might reveal new therapeutic targets to overcome infection with *Bb* by strengthening and supporting the host’s own immune system.

## Materials and Methods

### Mice

Female C57BL/6J (B6), BALB/cByJ (cBy), B6.129S2-Cd40lg^*tm1Imx*^/J (CD40L -/-), male or female B6.CB17-*Prkdc* ^*scid*^/SzJ (B6-SCID), and CBySmn.CB17-*Prkdc* ^*scid*^/J (BALB/c-SCID) mice were purchased from The Jackson Laboratory. B6-MyD88-/- breeding pairs were a generous gift from R. A. Flavell (Yale, CT). TS-1 mice on a BALB/c background, (breeder pairs kindly provided by A. Caton, The Wistar Institute) express a transgenic T cell receptor specific for influenza virus A/Puerto Rico/34/8 (A/PR8, H1N1) hemagglutinin (HA) peptide residues 110 to 119 presented on I-E^d^ [44]. All mice were maintained in microisolator cages under specific pathogen free conditions (screened for 17 common mouse pathogens, list available upon request).

### Ethics statement

This study was carried out in strict accordance with the recommendations in the Guide for the Care and Use of Laboratory Animals of the National Institutes of Health. All protocols involving animals were approved by the Animal Use and Care Committee at UC Davis (Permit Number: #15330).

### Infection of SCID mice, Borrelia burgdorferi, and recombinant proteins

For host-adaptation of spirochetes, SCID mice were infected by subcutaneous injection of 1x10^4^ (B6-SCID) or 1x10^6^ (BALB/c-SCID) culture grown *Bb* cN40 spirochetes. *B*. *burgdorferi* sensu stricto strain cN40 was cultured in modified Barbour-Stoenner-Kelley II medium at 33°C to mid-log phase and enumerated with a Petroff-Hauser bacterial cell counting chamber (Baxter Scientific) prior to infection of SCID mice. Recombinant proteins from *Bb*-cN40 sequence were generated as previously described[20]. GenScript later produced recombinant DbpA.

### Infections, immunizations and antibiotic treatment

Mice were infected with host-adapted *B*. *burgdorferi* cN40 as previously described[20]. Briefly, spirochetes were first tissue-adapted in SCID mice for 2–5 weeks, and then ear tissue pieces containing tissue-adapted spirochetes were transplanted beneath the skin of the right hind leg of mice for study. For immunization with recombinant Arp (from strain N40, generated in house[45]), mice were injected subcutaneously with 25 μg of recombinant protein in alum (Imject Alum, Thermo Scientific) on both sides of the tail base on days 0, 14, and 28 and analyzed on day 42. For influenza immunization, mice were injected subcutaneously on the right side of the tail base with 500 hemagglutinating units (HAU) of A/PR8 (H1N1) in alum. For determination of serum protective capacity, 50 μL of immune serum was diluted to 100 μL with PBS and injected i.v. into the tail vein; mice were then infected intranasally with 120 PFU A/PR8 in 40 μL PBS, an amount that was previously determined to be a lethal dose for naïve mice. Virus was propagated in embryonated hen eggs, and HAU determined as previously described[46]. When stated, *Bb* infected mice were treated once daily for 10 days with 16 mg/kg ceftriaxone (Sigma, product number C5793) in 100 μL PBS.

### Flow cytometry

Single cell suspensions of lymphoid tissues were prepared as previously described[47]. Cells were stained at 2.5–5x10^7^ cells/mL as described previously [33]. The following antibodies were used at previously determined optimal concentrations, made in-house unless otherwise stated; all cells were first blocked with anti-FcR antibody (clone 2.4G2 at 5 μg/mL) for 15 min on ice and washed with staining medium. For T_FH_ and T_GC_ stains, cells were then stained with CXCR5-biotin (BD Biosciences) and CXCR4-allophycocyanin (APC) (BD Biosciences) at 37°C for 45 min and washed. Surface antigens were stained for 20 min on ice with streptavidin-QDot605 (Molecular Probes), ICOS-fluorescein isothiocyanate (FITC) (purified clone 7E.17G9 from BD Biosciences, conjugated in-house), CD19-Cy5PE (eBioscience), CD4-Cy5.5PE, and CD3 efluor780-APC (eBioscience). When indicated, anti-TS-1 T cell receptor-biotin (6.5–2) and CXCR5-PE (BD Biosciences) were substituted. For B cell stains, surface antigens were stained for 20 min on ice with the following conjugates: CD3-Pacific Blue, CD4-Pacific Blue, CD8α-Pacific Blue, F4/80-Pacific Blue, (those for were used as “dump”), CD38-FITC, CD19-Cy5PE, CD24-Cy5.5PE, CD138-APC (BD Biosciences), and CD45R-efluor780-APC (eBioscience). When indicated, biotinylated peanut agglutinin (PNA; Vector Laboratories), followed by streptavidin-QDot605, GL-7-FITC (BD Bioscience), CD38-PE, IgD-Cy7PE and FAS-APC were added/substituted. Cells were washed twice and stained with the Live/Dead fixable violet dead cell discriminator (Invitrogen) for 30 min on ice, washed and resuspended in staining medium for fluorescence-activated cell sorter (FACS) analysis. Data were collected on either a FACSAria (BD Biosciences) or LSRFortessa (BD Biosciences) instrument and analyzed with FlowJo software (Tree Star Inc.).

### Adoptive transfers

For adoptive transfer of memory B cells, single cell suspensions of individual spleens were prepared and red blood cells were lysed [47] for 60 sec, followed by 2 washes with staining buffer, and 3 washes with PBS. Cells were then resuspended to 1x10^8^ cells/mL in PBS and 1x10^7^ cells were injected intravenously in the tail veins of each recipient mouse. For adoptive transfer of TS-1 T cells, pooled cells were washed 3 times in PBS after magnetic cell separation, resuspended to 1x10^7^ cells/mL in PBS, and 1x10^6^ cells were injected intravenously in the tail vein of each mouse.

### Memory B cell detection

After 45 days of *Bb* infection, mice were treated with antibiotics as described above. Donor mice were euthanized one day after the last antibiotic treatment and cells from each donor spleen were adoptively transferred into a naïve mouse. After 6–8 weeks, recipients were challenged with 50 μg recombinant Arp protein in 100 μL PBS intravenously. Four days later, antibody-secreting cells were quantified from spleens by ELISPOT.

### ELISPOT

96-well plates were coated overnight with DbpA (Genscript, 1 μg/mL), Arp (2.5 μg/mL), or influenza A/PR8 (1000 HAU/mL) in PBS. After blocking, single cell suspensions of spleens or lymph nodes were incubated in 2-fold serial dilutions beginning at 5x10^5^ cells per well for 18–20 hours in culture medium. Antigen-specific antibody spots were detected using biotinylated goat anti-mouse IgG or IgM, followed by streptavidin-horseradish peroxidase, and 10 min incubation with substrate. Spots were counted in each well on an AID iSpot Spectrum Reader (Advanced Imaging Devices GmbH). Mean antigen-specific ASC/1x10^6^ lymphocytes and SD for each tissue were calculated from all wells with countable spots.

### ELISA

As previously described [22], 96-well plates were coated with recombinant DbpA, Arp, OspC or BmpA in PBS overnight, washed and blocked for 1 hour, serial dilutions of serum incubated for 2–3 hours, and detected with biotinylated goat anti-mouse IgG or IgM, followed by streptavidin-horseradish peroxidase, and finally incubated for 20 min with substrate. Absorbance was read on a SpectraMax M5 (Molecular Devices) at 450 nm with a 595 nm reference wavelength. Serum from 120 days post *B*. *burgdorferi* infection served as a standard to determine relative units/mL of *B*. *burgdorferi*-specific antibody.

### Magnetic cell separation

To enrich for CD4+ T cells from TS-1 mice, unwanted cells were depleted by magnetic cell separation. Spleen single cell suspensions from naïve TS-1 mice were labeled with biotinylated antibodies against CD8α, CD19, CD11b, and CD49b (Biolegend). Cells were then labeled with anti-biotin MicroBeads (Miltenyi Biotec) and separated with an autoMACS instrument (Miltenyi Biotec). Purities ranged from 91–95% as determined by FACS analysis using CD4-APC or CD4-PE staining on a small number of cells.

### Histology

Histological sections were prepared as previously described [33] with the following changes: sets of three 5 μm serial sections were cut from frozen lymph nodes and dried onto three individual slides (i.e. slide 1, 2 and 3), then approximately 200 μm deeper into the tissue this procedure was repeated, for a total of 4 sections per slide. This resulted in three slides, eachhaving 4 sections spanning approximately 800 μm of tissue. The serial sections from each depth could be stained with three different follicular dendritic cell markers. Two of these sets (spanning approximately 800–1600 μm of tissue; i.e. slides 1, 2, 3 and 4, 5, 6) were used for analysis. All slides were stained overnight at room temperature with CD4-efluor450 (clone RM4-5; eBiosciences) and one serial section of three was stained with FDC-M2-Biotin (ImmunoKontact) (i.e. slides 1 and 4), CD21/35-Biotin (clone 7G6; in house) (i.e. slides 2 and 5), or PNA-Biotin (Vector Labs) (i.e. slides 3 and 6). Slides were then washed and stained for 1–2 hours at room temperature with IgD-FITC (clone 11.26c; in house) and streptavidin-AlexaFluor594 (Invitrogen). Slides were then washed, and mounted in Fluoromount-G (SouthernBiotech). Slides were imaged with either an Olympus BX61 microscope and an Olympus DP72 color camera, or an Olympus VS110 Slide Microscope Scanner with an Olympus XM10 camera. Images collected from the BX61 microscope were processed using MetaMorph and ImageJ (NIH) software, and images from the Olympus VS110 were processed using OlyVIA and FIJI (NIH) software. Images shown in Fig 4A were collected from the Olympus VS110 at 20x magnification, aligned in OlyVIA software, and presented at 2.5x magnification to present the most comprehensive overview of the tissues, scale bars represent 500 μm. Sections were arranged on slides as described above such that groups of 4 sections could be stained, imaged, and displayed under the same conditions and display scale settings.

### Statistical analysis

All graphs were prepared and statistical analysis was performed with GraphPad Prism version 5 software (GraphPad Software).

## Supporting Information

S1 Fig FDC-M2 staining is induced strongly in immunized but not *B*. *burgdorferi*-infected mice. C57BL/6 mice were immunized with influenza virus in alum and/or infected with Bb as indicated. Draining lymph nodes were collected 10 days later and cryopreserved. Frozen sections were prepared and stained with anti-mouse IgD FITC and anti-FDC-M2 biotin and streptavidin-AlexaFluor594 as outlined in the main manuscript. Germinal centers within IgD positive follicles are identified as IgDlow/negative. Shown are images collected at 10x objective of FDC-M2 staining (red) overlayed with IgD (green, left column) and alone (white, right column). Influenza immunization (top row), but not Bb infection (bottom two rows), induced robust FDC-M2 staining. Thus, Bb-infection results in a lack of complement C4 deposition on FDC.
(PDF)
