# Supplementary material for: Suppression of Long-Lived Humoral Immunity Following Borrelia burgdorferi Infection
Source: PLoS Pathog. 2015 Jul 2;11(7):e1004976. doi: 10.1371/journal.ppat.1004976 (PMC4489802; doi:10.1371/journal.ppat.1004976)
Supplement: S1 Fig — C57BL/6 mice were immunized with influenza virus in alum and/or infected with Bb as indicated. Draining lymph nodes were collected 10 days later and cryopreserved. Frozen sections were prepared and stained with anti-mouse IgD FITC and anti-FDC-M2 biotin and streptavidin-AlexaFluor594 as outlined in the main manuscript. Germinal centers within IgD positive follicles are identified as IgDlow/negative. Shown are images collected at 10x objective of FDC-M2 staining (red) overlayed with IgD (green, left column) and alone (white, right column). Influenza immunization (top row), but not Bb infection (bottom two rows), induced robust FDC-M2 staining. Thus, Bb-infection results in a lack of complement C4 deposition on FDC. (PDF) [file ppat.1004976.s001.pdf]

Supplemental Information

Suppression of long-lived humoral immunity following *Borrelia burgdorferi* infection

Rebecca A. Elsner, Christine J. Hastey, Kimberly J. Olsen, and Nicole Baumgarth

Supplemental Figure 1

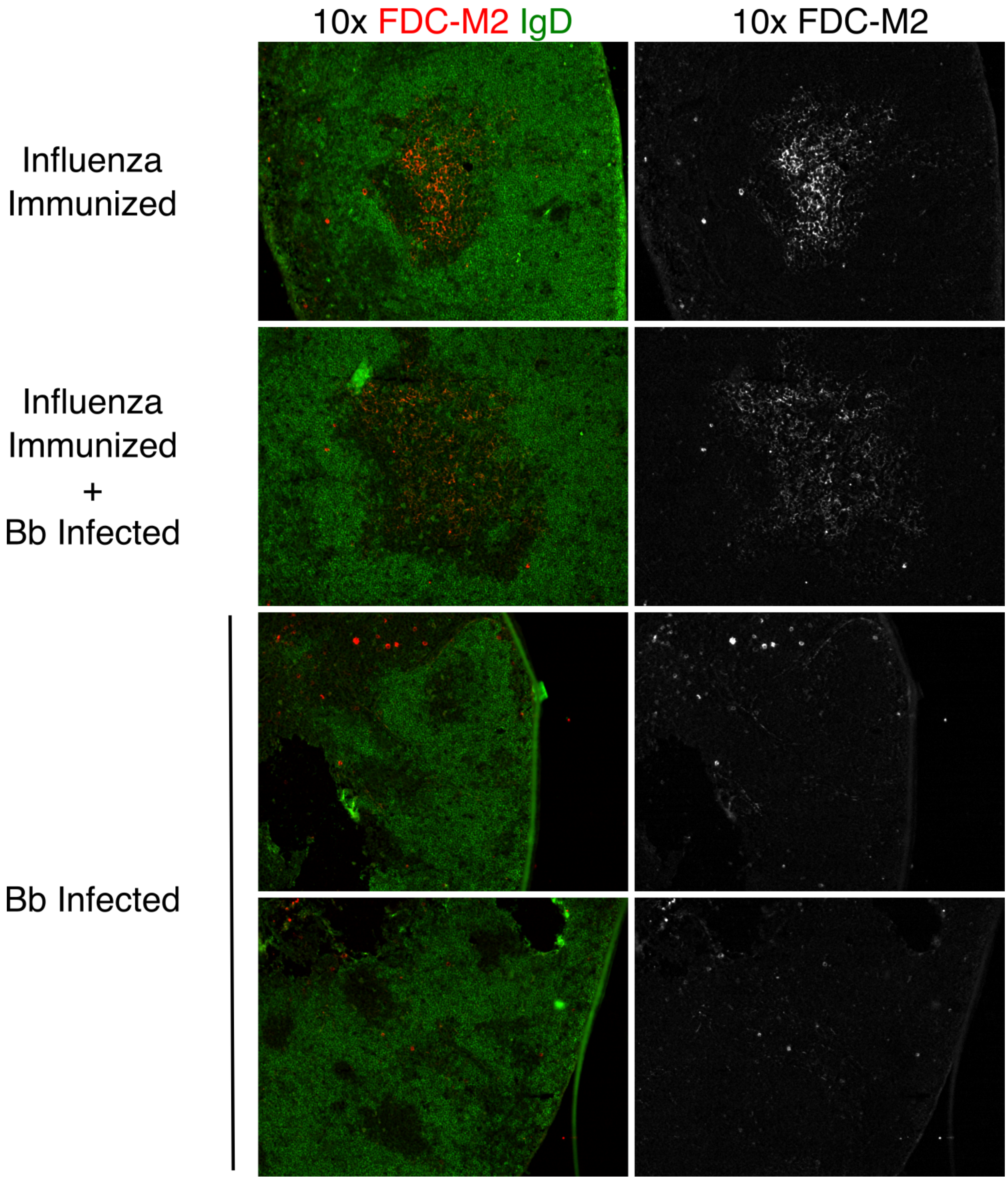

Supplemental Figure 1 FDC-M2 staining is induced strongly in immunized but not B. burgdorferi-infected mice C57BL/6 mice were immunized with influenza virus in alum and/or infected with Bb as indicated. Draining lymph nodes were collected 10 days later and cryopreserved. Frozen sections were prepared and stained with anti-mouse IgD FITC and anti-FDC-M2 biotin and streptavidin-AlexaFluor594 as outlined in the main manuscript. Germinal centers within IgD positive follicles are identified as IgDlow/negative. Shown are images collected at 10x objective of FDC-M2 staining (red) overlayed with IgD (green, left column) and alone (white, right column). Influenza immunization (top row), but not Bb infection (bottom two rows), induced robust FDC-M2 staining. Thus, Bb-infection results in a lack of complement C4 deposition on FDC.
